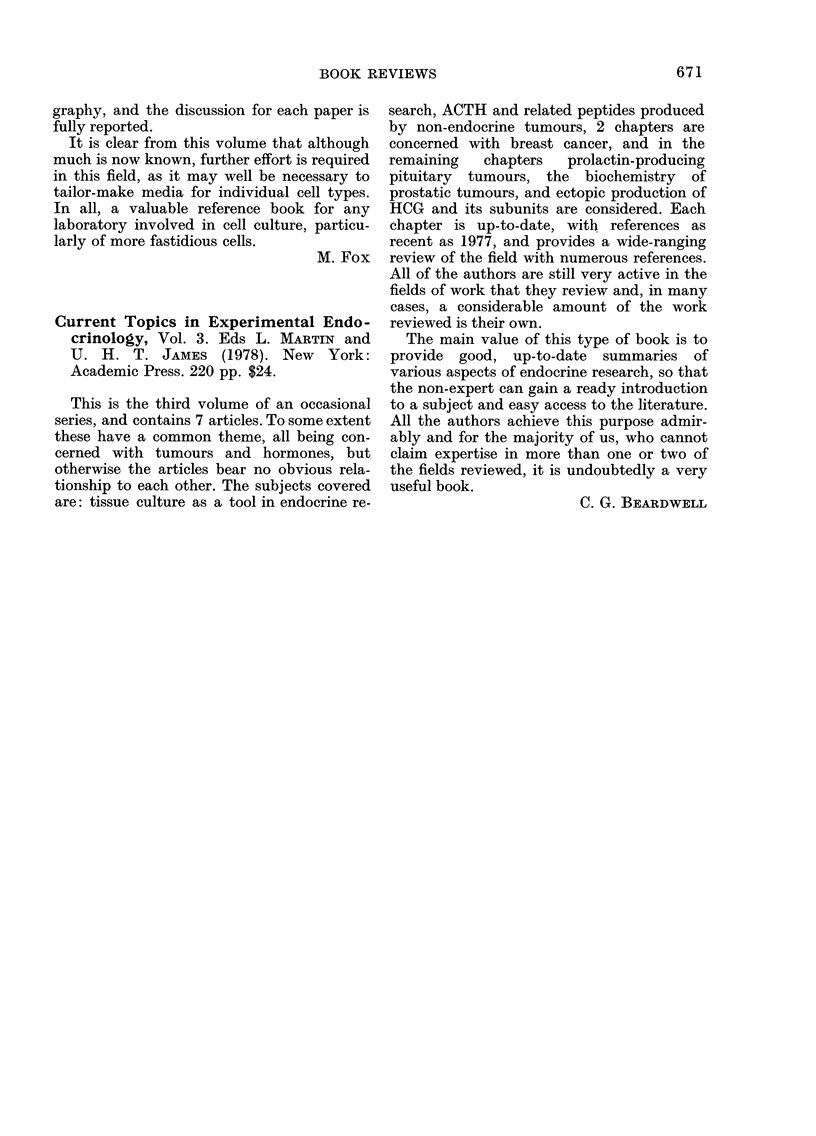# Current Topics in Experimental Endocrinology, Vol. 3

**Published:** 1979-10

**Authors:** C. G. Beardwell


**Current Topics in Experimental Endocrinology,** Vol. 3. Eds L. Martin and U. H. T. James (1978). New York: Academic Press. 220 pp. $24.

This is the third volume of an occasional series, and contains 7 articles. To some extent these have a common theme, all being concerned with tumours and hormones, but otherwise the articles bear no obvious relationship to each other. The subjects covered are: tissue culture as a tool in endocrine research, ACTH and related peptides produced by non-endocrine tumours, 2 chapters are concerned with breast cancer, and in the remaining chapters prolactin-producing pituitary tumours, the biochemistry of prostatic tumours, and ectopic production of HCG and its subunits are considered. Each chapter is up-to-date, with references as recent as 1977, and provides a wide-ranging review of the field with numerous references. All of the authors are still very active in the fields of work that they review and, in many cases, a considerable amount of the work reviewed is their own.

The main value of this type of book is to provide good, up-to-date summaries of various aspects of endocrine research, so that the non-expert can gain a ready introduction to a subject and easy access to the literature. All the authors achieve this purpose admirably and for the majority of us, who cannot claim expertise in more than one or two of the fields reviewed, it is undoubtedly a very useful book.

C. G. Beardwell